# Role of First Pass (FP) and delayed enhancement in functional recovery assessment after Acute Myocardial Infarction (AMI)

**DOI:** 10.1186/1532-429X-11-S1-P121

**Published:** 2009-01-28

**Authors:** Antonio Bernardini, Luigi Natale, Agostino Meduri, Carlo Liguori, Antonella Lombardo, Lorenzo Bonomo

**Affiliations:** 1 "G. Mazzini" Hospital – ASL Teramo, Teramo, Italy; 2 Università Cattolica del Sacro Cuore – Policlinico A. Gemelli, Rome, Italy

**Keywords:** Public Health, Myocardial Infarction, Acute Myocardial Infarction, Acute Myocardial Infarction, Functional Recovery

## Purpose

To define contrast enhanced MRI role in functional recovery prediction after acute myocardial infarction.

## Materials and methods

46 patients with first AMI (65 ± 8 yrs., 39 anterior, 4 inferior, 3 lateral) underwent cardiac MRI after primary PTCA 5 to 7 days after onset. MRI Protocol: SA Cine (FIESTA), 8–10 slices; SA first pass (FGRE-ET) (iv 0.1 mmol/kg Gd-DTPA, 3 mL/s), 3 slices; SA delayed imaging (IR-prep FGRE), 8–10 slices, 15 mins after 0,2 mmol/Kg Gd-DTPA. FP and DE were evaluated together (736 segments) as: normal FP, DE absent or up to 50% of transmural extension (TME) (Pattern 1); delayed hyperenhancement >50% without FP hypoenhancement (Pattern 2a); delayed hyperenhancement >50% with FP hypoenhancement (Pattern 2b); hypoenhancement both at FP and DE(Pattern 3). 828 segments out of first-pass slices were classified on the basis of DE as normal or hyperenhanced 50% TME (= pattern 2) and hypoenhanced (= pattern 3). Patterns 2, 2a, 2b and 3 were considered non viable. Six months follow up MRI assessed functional recovery as improvement of segmental WMSI.

## Results

Pattern 1: 1167 segments, functional recovery in 1065 (91,3%). Pattern 3: 132 segments, recovery in 9 (6,8%) Pattern 2: 265 segments, recovery in 78 (29,4%). Pattern 2a showed recovery in 23 segments out of 42 (54,8%); Pattern 2b did not recover in 61 out of 80 (76,3%).

## Conclusion

Patterns 1 and 3 respectively identify viable and non viable tissue. Pattern 2 is less specific early after AMI; the presence of FP hypoenhancement is often associated with failed recovery.

